# A novel hybrid CNN-transformer model for arrhythmia detection without R-peak identification using stockwell transform

**DOI:** 10.1038/s41598-025-92582-9

**Published:** 2025-03-06

**Authors:** Donghyeon Kim, Kyoung Ryul Lee, Dong Seok Lim, Kwang Hyun Lee, Jong Seon Lee, Dae-Yeol Kim, Chae-Bong Sohn

**Affiliations:** 1 Department of Defense Acquisition Program, Kwangwoon University, Seoul, 01897 Republic of Korea; 2 HolmesAI, Headquarters, Daegu, 41260 Republic of Korea; 3 AI Research Center, HolmesAI, Seoul, 03185 Republic of Korea; 4 Department of Artificial Intelligence, Kyungnam University, Changwon-si, Gyeongsangnam-do 51767 Republic of Korea

**Keywords:** Arrhythmia classification, Stockwell transform, ECG signal processing, Hybrid CNN-transformer model, Arrhythmias, Computational biology and bioinformatics, Cardiology

## Abstract

This study presents a novel hybrid deep learning model for arrhythmia classification from electrocardiogram signals, utilizing the stockwell transform for feature extraction. As ECG signals are time-series data, they are transformed into the frequency domain to extract relevant features. Subsequently, a CNN is employed to capture local patterns, while a transformer architecture learns long-term dependencies. Unlike traditional CNN-based models that require R-peak detection, the proposed model operates without it and demonstrates superior accuracy and efficiency. The findings contribute to enhancing the accuracy of ECG-based arrhythmia diagnosis and are applicable to real-time monitoring systems. Specifically, the model achieves an accuracy of 97.8% on the Icentia11k dataset using four arrhythmia classes and 99.58% on the MIT-BIH dataset using five arrhythmia classes.

## Introduction

An electrocardiogram (ECG) is a vital recording method of the heart’s electrical activity, used for the diagnosis and management of various cardiovascular diseases^1^. Abnormalities in external heart beating rhythms, such as arrhythmia, require early detection to improve patient outcomes. Standard diagnostic practices depend on the skills of medical practitioners. However, various ECG signals are often complex and may vary even within a single patient. Leading to longer acquisition times and introducing subjectivity due to personal differences in perception^2^. The dependency on practitioners has increased the need for developing automated diagnostic systems. This has led to extensive research on ECG signals using deep learning approaches^3^.

Recently, deep learning techniques, specifically Convolutional Neural Networks (CNNs), have been employed to extract local features from ECG signals to improve the accuracy of arrhythmia detection^4^. Nevertheless, while CNNs are effective at capturing spatial patterns in signals, they struggle with and are bad at learning temporal sequences or long-term dependencies that time-series data, such as ECG signals, contain^5^. To overcome this limitation, Transformer models with their powerful attention mechanisms have been recognized as effective tools for analyzing time-series data. Particularly due to their ability to handle their ability to handle long-range dependencies^6^.

The fact that ECG signals contain not only time but also frequency information, needs to be taken into account for accurately capturing abnormal heart activities. The extracting feature methods from ECG signals, e.g., wavelet transform^7^, are generally done using time-frequency analysis techniques. While wavelet-based methods are useful for analyzing local time and frequency properties of signals, they may suffer from limited resolution. In this study, we tackle these challenges to extract time-frequency features from ECG signals by utilizing Stockwell Transform (S-transform). This is effective in extracting features from ECG signals because it can adaptively capture variations in both time and frequency^8^.

In this study, we propose a classification model that first extracts features from ECG signals using the S-transform and then applies these features to a hybrid deep learning model combining CNN and Transformer. The CNN component learns local patterns in the signals to effectively extract important characteristics from the ECG data, while the Transformer component captures long-term dependencies from these features. This hybrid approach aims to overcome the limitations of existing deep learning-based models and enhance the accuracy of some arrhythmia classification, like premature beats. Since the S-transform represents data in the frequency domain, it offers an advantage over traditional methods that require R-peak detection, which can fail in certain arrhythmias. By not relying on R-peak detection, the proposed method can address cases where R-peaks are not detectable. Furthermore, the proposed model demonstrates superior performance in terms of accuracy compared to existing CNN-based arrhythmia classification models.

The subsequent sections of this paper are organized as follows: “Literal Reviews” presents the previous machine learning and deep learning approaches, “Material and Methods“ explains the proposed model structure, “Experiments” presents experimental details, “Result and Discussion” discusses the results, and “Conclusion” concludes the study.

## Literal reviews

ECG signals are highly complex and noise-sensitive, necessitating robust preprocessing techniques to ensure accurate analysis^9^. Traditional ECG preprocessing methods primarily focus on noise reduction and artifact suppression, utilizing low-pass, high-pass, and band-pass filters to mitigate power line interference, baseline wander, and muscle artifacts^10^. In addition to linear filtering, nonlinear denoising approaches, such as Wavelet Transform, have demonstrated effectiveness in preserving time-frequency localization and capturing signal nonlinearity^11^.

ECG classification has traditionally relied on machine learning models, including K-Nearest Neighbors (KNN), Support Vector Machines (SVM), and Random Forest^12^. These methods depend on handcrafted feature extraction, where techniques such as Wavelet Transform and Principal Component Analysis (PCA) play a crucial role in enhancing classification performance^13^. However, the need for manual feature engineering limits scalability and adaptability in real-world applications.

In recent years, deep learning approaches have gained prominence in ECG classification. CNN-based models effectively extract local features from ECG signals and have been widely adopted for automatic arrhythmia detection^4^. Unlike traditional methods, CNNs eliminate the need for explicit feature extraction, allowing models to learn relevant signal representations during training. However, as ECG signals exhibit sequential dependencies, LSTM-based architectures have been explored to capture long-term temporal correlations^14^. More recently, Transformer models have demonstrated superior performance in time-series analysis, outperforming both CNNs and LSTMs in modeling long-range dependencies^15^.

This study introduces a hybrid framework that integrates S-transform-based feature extraction with a CNN-Transformer architecture to leverage both local and global signal characteristics. The S-transform facilitates time-frequency representation, enabling more comprehensive feature extraction, while the combined CNN-Transformer model enhances classification efficiency. By addressing the limitations of conventional machine learning and standalone deep learning models, the proposed methodology aims to improve arrhythmia classification accuracy and robustness.

## Materials and methods

### Dataset

In this study, the MIT-BIH Arrhythmia dataset and Icentia11k dataset were used for ECG signal arrhythmia classification. The MIT-BIH Arrhythmia Database is among the most extensively utilized ECG datasets, containing data from 48 patients^16^. Due to its inclusion of various types of arrhythmias, it serves as a benchmark for evaluating ECG analysis algorithms. Each record comprises two leads recorded over a 30-minute duration with a sampling frequency of 360 Hz. This dataset has been established as the standard for arrhythmia classification research, demonstrating its reliability across numerous studies. Arrhythmias in the MIT-BIH dataset can be categorized into N, S, V, Q, and F symbols, corresponding to the AAMI classes. By including well-defined and frequently observed arrhythmias, the MIT-BIH dataset provides a standardized framework for assessing the robustness and accuracy of classification models. The Icentia11k dataset is a comprehensive collection of ECG data recorded from more than 11,000 patients in hospitals in Quebec, Canada, including long-term Holter monitoring records^17^. This dataset not only includes common arrhythmias but also includes a variety of ECG patterns that can occur in daily life, making it very suitable for real-world arrhythmia classification tasks. The provision of long-term continuous data offers significant advantages for analyzing extended ECG patterns and training robust classification models. The Icentia11k dataset is categorized into four classes: N, S, V, and Q, with the class. The dataset’s class distribution and description are shown in Table 1.

**Table 1:** Class distribution and data description in Icentia 11k and MIT-BIH datasets.

Dataset	Class	Description	Count
MIT-BIH	N	NOR (Normal Beat) / LBBB (Left Bundle Branch Block) / RBBB (Right Bundle Branch Block) / AE (Atrial Escape Beat) / NE (Nodal Escape Beat)	3366
	S	AP (Atrial Premature beat) / aAP (Aberrated atrial premature beat) / SP (Supra ventricular premature beat) / NP (Nodal premature beat)	203
	V	PVC (Premature Ventricular Contraction) / VE (Ventricular Escape beat)	922
	F	Fvn (Fusion of normal & ventricular beat)	15
	Q	P (Paced beat) / fPN (Fusion of paced & normal beat) / U (Unclassified beat)	534
Icentia 11k	N	Normal	2,061,141,216
	S	PAC: Premature or ectopic supra-ventricular beat, premature atrial contraction	19,346,728
	V	PVC: Premature ventricular contraction	17,203,041
	Q	Undefined: Unclassifiable beat	646,364,002

The two datasets used in this study, MIT-BIH and Icentia11k, differ in their ECG lead configurations. The MIT-BIH dataset utilizes signals from Modified Lead I, whereas the Icentia11k dataset employs signals from Lead II. These differences in lead configuration result in variations in ECG signal characteristics, such as amplitude, phase, and the representation of specific arrhythmias. Differences in the heart’s electrical axis orientation can cause significant morphological variations between the two leads. By leveraging both datasets, this study provides an opportunity to evaluate whether the proposed model can extract robust and generalizable features regardless of the lead configuration. The MIT-BIH dataset includes an additional label, Fusion (F), which is not present in the Icentia11k dataset. The Fusion class represents a mixture of ventricular and normal beats, capturing transitional and ambiguous arrhythmic states. This class provides unique advantages for evaluating the model’s ability to handle complex and overlapping arrhythmia patterns.

In this study, the MIT-BIH Arrhythmia dataset was augmented using the Synthetic Minority Over-sampling Technique^18^ (SMOTE) to address the class imbalance issue. SMOTE was applied to generate synthetic samples for underrepresented classes, ensuring a more balanced dataset for model training. This technique allowed the model to learn more effectively from minority classes and improved the overall classification performance.

### Preprocessing steps

In our study, the preprocessing of raw ECG data is conducted through a structured pipeline consisting of several stages, including 50-second windowing, noise removal using a 30Hz low-pass filter, computational reduction via downsampling to 100Hz, baseline correction by detrending, additional segmentation with 10-second windowing, outlier removal using the 75% interquartile range (IQR), Min-Max normalization, and the S-transform for time-frequency analysis.

**Fig. 1:**
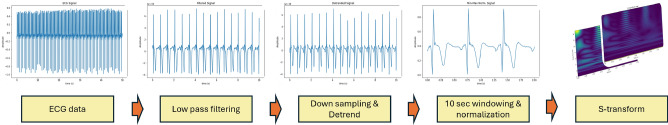
The preprocessing steps used in this study.

Figure 1 delineates the comprehensive preprocessing stages employed in this study. The preliminary step of preprocessing involves partitioning the raw ECG signal into 50-second segments. This windowing strategy was chosen to reduce computational complexity and facilitate more manageable data processing in subsequent stages. By dividing the continuous ECG data into smaller, distinct time intervals, memory utilization, and computational efficiency are significantly enhanced, thus establishing a more effective and streamlined preprocessing workflow.

In the second step, we implemented noise reduction using a 30Hz low-pass filter to mitigate prevalent sources of interference in ECG recordings. ECG signals frequently exhibit interference stemming from diverse sources, such as power line disturbances at 50Hz or 60Hz, which can substantially impact signal quality and analysis results^1^. To counter these influences, a 30Hz low-pass filter was utilized to eliminate higher-frequency noise components while preserving the essential information necessary for precise ECG analysis. This filter guarantees that the signal remains devoid of common artifacts associated with power sources, muscular contractions, and other extraneous interferences.

The third step involved downsampling the data to 100 Hz. Downsampling is a commonly used method in ECG signal analysis that aids in reducing data size while retaining the critical features necessary for arrhythmia classification^16^. Previous studies on ECG classification have shown that a downsampling rate of 100 Hz is adequate for preserving the essential diagnostic features of the ECG signal while reducing the computational load. By decreasing the sampling rate, our goal is to maintain an optimal balance between computational efficiency and the retention of diagnostically relevant information. We implemented a baseline correction technique using the advanced detrending method proposed by Tarvainen et al^19^. in their study on HRV (heart rate variability) analysis. ECG signals frequently display baseline wandering caused by subject movement or electrode instability. This wandering can introduce significant variability, negatively impacting the effectiveness of subsequent feature extraction and classification tasks. The detrending method is employed to remove these low-frequency trends, thereby improving the reliability of the ECG signal for further analysis.

The fourth step involves further dividing the data into 10-second intervals to ensure consistent time segments for analysis. After segmentation, we conducted outlier removal using the 75th percentile interquartile range (IQR) method, which aids in filtering out extreme values that could distort the model’s learning process. Following the outlier removal, Min-Max normalization was implemented to standardize the data between 0 and 1. This normalization step is essential to ensure that all input data have a consistent range, which facilitates the training process and helps prevent issues related to differing scales among features, ultimately contributing to improved model convergence.

Finally, we utilized the S-transform on the ECG data to convert the signals into the time-frequency domain. The mathematical expression for the S-transform is depicted in Equation 1.

1 $$\begin{aligned} S(x, y, f) = \int ^\infty _{-\infty } \int ^\infty _{-\infty } f(x', y') g(x-x', y-y', \sigma (x', y')) e^{-j 2\pi (u x' + v y')} dx' dy' \end{aligned}$$

The function *f*(*x*, *y*) is convolved with a Gaussian window $g(x-x', y-y', \sigma (x', y'))$, where $\sigma (x', y')$ represents the scale parameter controlling the time-frequency localization. This process results in a time-frequency representation *S*(*x*, *y*, *f*) that captures both the spatial and spectral characteristics of the input signal.

**Fig. 2:**
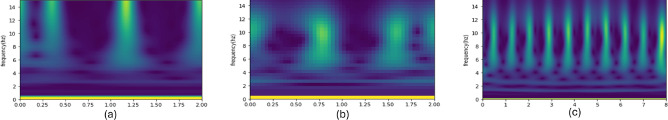
Impact of sampling frequency and signal duration on the time-frequency resolution of the S-transform : (**a**) 100 Hz, 2-second signal; (**b**) 25 Hz, 2-second signal; (**c**) 25 Hz, 8-second signal.

The S-transform provides a localized time-frequency representation by combining the advantages of the Fourier and Wavelet transforms^8^. Figure 2 illustrates the inherent trade-off between time and frequency resolution. For the same 2-second ECG signal, Fig. 2a (100 Hz sampling) achieves higher temporal resolution, capturing rapid signal changes with finer time-domain details. In contrast, Fig. 2b (25 Hz sampling) sacrifices temporal resolution but preserves broader spectral characteristics. When the number of samples is held constant, as in Fig. 2a versus c (25 Hz sampling over a longer duration), lower sampling frequencies enhance frequency resolution, improving spectral detail at the cost of temporal precision.

This adaptability is particularly beneficial for ECG analysis, where distinguishing fine temporal variations and spectral features is critical for arrhythmia classification. Additionally, the S-transform obviates the need for conventional feature extraction techniques such as R-peak detection, commonly employed in ECG analysis^20^. By concurrently providing temporal and spectral information, the S-transform facilitates comprehensive ECG signal analysis, capturing intricate time-frequency dynamics essential for detecting pathological conditions.

To further optimize the effectiveness of the S-transform in ECG analysis, we constrained the frequency range to 0-15 Hz. This decision was based on the occurrence of muscle noise and other artifacts, such as electrical surgical unit (ESU) interference, typically manifesting at frequencies higher than 15 Hz^21^. By limiting the frequency response to this range, our aim was to minimize the impact of muscle noise while preserving the essential frequency components required for accurate arrhythmia detection. This approach ensures that the signal retains its fundamental characteristics for classification while mitigating noise from external sources.

**Fig. 3:**
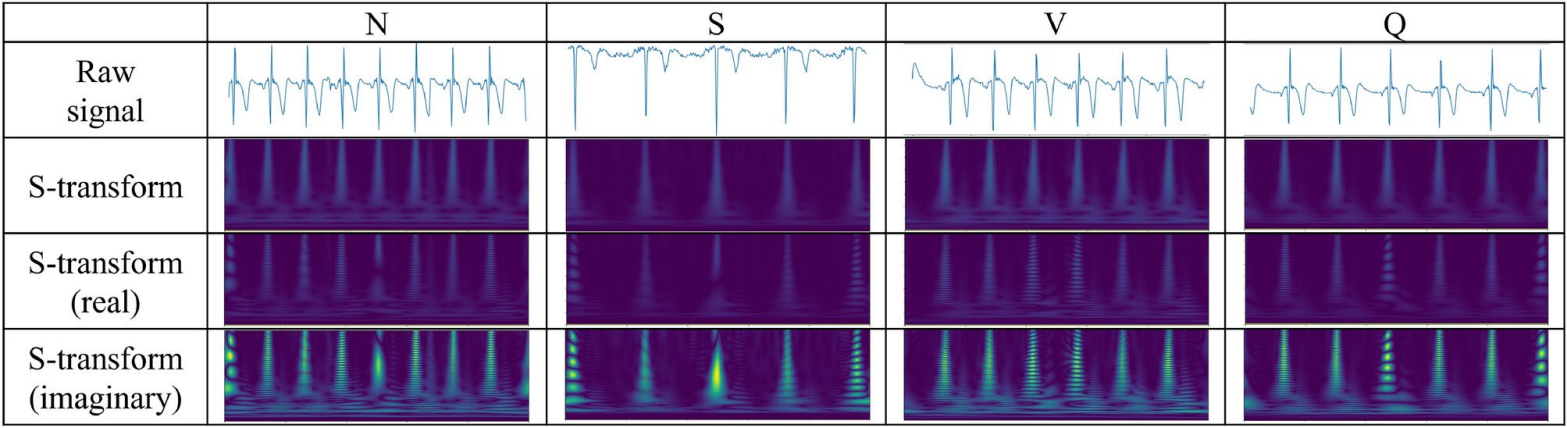
2D S-transform results of Icentia11k dataset.

In preparation for input into the deep learning model, the S-transformed data was decomposed into its real and imaginary components. These components were then utilized as inputs to the neural network, thus retaining comprehensive information pertaining to the characteristics of the ECG signal. Through harnessing the time-frequency representation offered by the S-transform, the suggested deep learning model can more effectively discern pertinent features for the detection of arrhythmia, ultimately resulting in improved model performance and accuracy.

### Arrhythmia classification on hybrid ResNextNet-transformer

This study proposes a hybrid model integrating ResNeXtNet and a Transformer encoder for ECG-based arrhythmia classification, designed to capture both local and long-range dependencies^22^. The input ECG signals are transformed into time-frequency representations using the S-transform, which combines the advantages of the Fourier and Wavelet transforms.

As illustrated in Fig. 3, this representation enables a detailed analysis of temporal variations and frequency components, making it well-suited for modeling the dynamic nature of ECG signals, including heart rate fluctuations. Unlike conventional methods that rely on R-peak detection, the S-transform extracts comprehensive time-frequency information directly, allowing the model to analyze global signal dynamics rather than focusing on specific fiducial points.

**Table 2:** The proposed network architecture. (a) proposed hybrid model. (b) ResNextBlock with SE module.

Configuration	Output shape
(a)	
Feature extractor (ResNeXt)	
Conv($7\times 7$, s=2, 64ch) $\rightarrow$ MaxPool($3\times 3$, s=2)	[*B*, 64, *H*/4, *W*/4]
ResNeXt Block $\times 2$ (64 $\rightarrow$ 64)	[*B*, 64, *H*/4, *W*/4]
ResNeXt Block $\times 2$ (64 $\rightarrow$ 128, s=2)	[*B*, 128, *H*/8, *W*/8]
ResNeXt Block $\times 2$ (128 $\rightarrow$ 256, s=2)	[*B*, 256, *H*/16, *W*/16]
ResNeXt Block $\times 2$ (256 $\rightarrow$ 512, s=2)	[*B*, 512, *H*/32, *W*/32]
Conv($1\times 1$, 512 $\rightarrow$ 512)	[*B*, 512, *H*/32, *W*/32]
Transformer encoder	
Reshape: $[B, 512, H/32, W/32] \rightarrow [B, T, 512]$	[*B*, *T*, 512]
Learnable 2D Positional Encoding	[*B*, *T*, 512]
Transformer Block $\times 4$ (heads=8,FFN=2048, d=0.2)	[*B*, *T*, 512]
Residual Connection (Skip connection)	[*B*, *T*, 512]
Mean Pooling	[*B*, 512]
Classification head	
Linear(512 $\rightarrow$ 256)	[*B*, 256]
LayerNorm(256)	[*B*, 256]
Linear(256 $\rightarrow$ num_classes)	[*B*, num_classes]

Layer	Configuration
(b)	
Conv2d($1\times 1$, bottleneck)	*in_planes* → *group_width*
Grouped Conv2d($3\times 3$)	*group_width* → *group_width*
	$$s=stride$$
Conv2d($1\times 1$, restore)	*group_width* → *out_planes*
SE Block	Reduction Ratio = 16
Stochastic Depth	drop_prob=0.2
Residual Connection	Enabled

The proposed network architecture, detailed in Table 2, integrates a ResNeXt-based CNN^23^ for feature extraction and a Transformer encoder for sequence modeling. This hybrid structure effectively captures local time-frequency representations and long-range dependencies in ECG signals.

As summarized in Table 2(a), the feature extraction stage utilizes ResNeXt blocks with grouped convolutions and $1\times 1$ bottlenecks to enhance computational efficiency. The Squeeze-and-Excitation (SE) mechanism dynamically recalibrates channel-wise attention, emphasizing salient features while suppressing noise. Extracted feature maps are then compressed via a $1\times 1$ convolution and reshaped into a sequential representation, where a learnable 2D positional encoding preserves spatial correlations.

The Transformer encoder, comprising four layers, processes these embeddings to model temporal dependencies. Each layer consists of Multi-Head Self-Attention (8 heads), a Feedforward Network (2048 dimensions), Dropout (0.2), and LayerNorm^24^, with residual connections ensuring stability. Mean pooling aggregates the encoded sequence into a compact representation, which is subsequently passed through fully connected layers with LayerNorm for classification.

As shown in Table 2(b), the ResNeXt block incorporates stochastic depth regularization (drop probability = 0.2) to mitigate overfitting, while the residual pathway facilitates gradient flow. By combining local feature extraction (ResNeXt) and global sequence modeling (Transformer), the model efficiently captures short-term variations and long-range temporal dependencies, leading to enhanced robustness and generalization in arrhythmia classification.

### Experiments

#### Experimental setup

This study optimized multiple hyperparameters to train the proposed hybrid model for ECG arrhythmia classification. The model was trained using the RAdam optimizer (learning rate = 3e-4), with mixed-precision training enabled via PyTorch’s GradScaler for enhanced GPU efficiency^25^. To mitigate class imbalance and enhance robustness, Focal Loss was employed instead of standard cross-entropy^26^.

The input data comprised S-transform-derived time-frequency representations. Batch sizes (64, 256) were explored, and training proceeded for up to 200 epochs with an early stopping criterion (patience = 15) to prevent overfitting. Various learning rate schedulers, including CosineAnnealingWarmRestarts and ExponentialLR, were evaluated to ensure stable convergence^27,28^.

The hybrid architecture integrated a ResNeXt-based CNN with SE modules, followed by a Transformer encoder for capturing long-term dependencies. Optimized hyperparameters contributed to improved classification performance, assessed via confusion matrices and F1-scores. The final model weights were saved for reproducibility, and all model definitions and training scripts are available in the referenced repository.

### Ablation study

#### Preprocessing strategies for reducing ECG artifacts in deep learning models

**Fig. 4:**
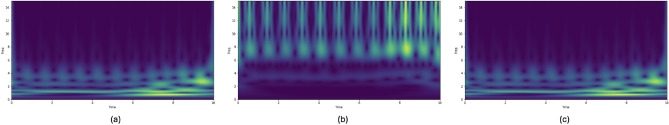
Comparison of S-transform features under different preprocessing conditions.

Artifacts in ECG signals pose significant challenges for deep learning-based arrhythmia classification. These artifacts, including baseline wander, power line interference, and high-frequency noise, distort ECG features and increase misclassification risk. To mitigate these distortions, Low-Pass Filtering (LPF) and Detrending are commonly applied before extracting time-frequency representations via the Stockwell Transform (S-transform).

Figure 4a compares a signal without Detrending to one with Detrending applied. The results show that Detrending effectively corrects baseline drift and plays a crucial role in stabilizing long-term signal variations.

Figure 4b compares a signal without LPF to one with LPF applied. LPF is applied before the S-transform to remove high-frequency components and refine the signal; however, its effect remains relatively minor and does not significantly alter the overall signal. Nonetheless, residual high-frequency components can cause phase instability, making the application of LPF necessary. By eliminating unnecessary high-frequency components before performing the S-transform, LPF helps extract more consistent spectral features in the frequency domain.

Figure 4c compares a signal without both LPF and Detrending to one with full preprocessing applied. This comparison highlights that Detrending plays a crucial role in correcting baseline variations and ensuring overall signal stability, with most filtering effects occurring at this stage. Notably, since 15 Hz was set as the upper frequency limit (Fmax) in this study, maintaining structural stability in the low-frequency range becomes an essential factor in preprocessing.

Overall, in this study, 15 Hz was set as the upper frequency limit (Fmax), ensuring that most high-frequency components were removed. As a result, baseline drift became the dominant issue, making Detrending the most impactful preprocessing step. However, some residual phase information (e.g., power line interference) remains, leading to potential high-frequency artifacts. To address this, LPF is essential for stabilizing the phase of remaining high-frequency components. Therefore, the optimal preprocessing strategy is to use Detrending for baseline drift removal and LPF to ensure phase stability in the filtered signal.

#### Evaluation of contributions: CNN, transformer, and S-transform

**Table 3:** Comparative study of CNN, transformer, and S-transform contributions to model performance (MIT-BIH and Icentia 11k).

Model	CNN		1D Transformer		2D CNN		2D Transformer		2D hybrid model	
	MIT-BIH	Icentia 11k	MIT-BIH	Icentia 11k	MIT-BIH	Icentia 11k	MIT-BIH	Icentia 11k	MIT-BIH	Icentia 11k
Overall Acc (%)	31.73	47.33	67.26	57.23	99.41	96.19	80.81	60.81	99.58	97.80
N										
F1	0.3289	0.3519	0.6696	0.5962	0.9881	0.9638	0.6782	0.5814	0.9896	0.9792
Se	0.4065	0.3417	0.6884	0.6924	0.9852	0.9749	0.6379	0.6333	0.9881	0.9913
PPV	0.2762	0.3628	0.6516	0.5235	0.9910	0.9530	0.7239	0.5374	0.9910	0.9674
S										
F1	0.0220	0.5736	0.6400	0.6615	0.9956	0.9758	0.7334	0.7906	0.9970	0.9707
Se	0.0119	0.4591	0.6190	0.5932	1.0000	0.9699	0.7410	0.7926	0.9970	0.9593
PPV	0.1428	0.7703	0.6624	0.7475	0.9912	0.9817	0.7259	0.7886	0.9970	0.9823
V										
F1	0.1203	0.5391	0.4250	0.6657	0.9896	0.9610	0.7001	0.6628	0.9926	0.9857
Se	0.0801	0.5936	0.3026	0.6877	0.9881	0.9610	0.7032	0.6777	0.9941	0.9880
PPV	0.2410	0.4938	0.7132	0.6451	0.9910	0.9610	0.6970	0.6485	0.9911	0.9834
Q										
F1	0.4360	0.4429	0.6849	0.3473	0.9970	0.9471	0.9255	0.3710	1.0000	0.9763
Se	0.6261	0.5010	0.8189	0.3156	0.9970	0.9419	0.9584	0.3287	1.0000	0.9733
PPV	0.3343	0.3968	0.5884	0.3860	0.9970	0.9524	0.8947	0.4260	1.0000	0.9792
F										
F1	0.4122	–	0.8521	–	1.0000	–	0.9912	–	1.0000	–
Se	0.4613	–	0.9345	–	1.0000	–	1.0000	–	1.0000	–
PPV	0.3725	–	0.7830	–	1.0000	–	0.9824	–	1.0000	–
Kappa	0.1465	0.2978	0.5908	0.4297	0.9926	0.9492	0.7601	0.4774	0.9948	0.9707
MCC	0.1559	0.3013	0.5988	0.4330	0.9926	0.9493	0.7605	0.4791	0.9948	0.9707

Table 3 summarizes the contributions of CNN, Transformer, and S-transform in ECG arrhythmia classification across the MIT-BIH and Icentia11k datasets.

Using raw 1D ECG signals, the 1D CNN based on TCN^29^ exhibited poor performance, achieving 31.73% accuracy (MIT-BIH) and 47.33% (Icentia11k). This outcome is attributed to the CNN’s reliance on local temporal features, which proved insufficient for capturing meaningful ECG relationships. The lack of explicit frequency-domain information, combined with arbitrary signal windowing (e.g., P wave, QRS complex, or T wave starting at varying locations), hindered pattern consistency.

The 1D Transformer improved classification, achieving 67.26% (MIT-BIH) and 57.23% (Icentia11k). While its self-attention mechanism effectively captured long-range dependencies, the absence of spectral information limited its feature extraction capabilities. A substantial performance boost was observed when ECG signals were transformed into 2D time-frequency representations using the S-transform. The 2D CNN with residual blocks achieved 99.41% (MIT-BIH) and 96.19% (Icentia11k), demonstrating the advantage of integrating temporal and spectral representations. By leveraging residual connections, the model captured complex hierarchical features while maintaining robustness to signal variations, such as lead configurations and noise^30^. The 2D Transformer, trained on S-transformed signals, reached 80.81% (MIT-BIH) and 60.81% (Icentia11k). Despite its effectiveness in modeling global dependencies, its inability to capture localized ECG features likely contributed to performance limitations. The 2D Hybrid Model, combining CNNs and Transformers, outperformed all architectures, achieving 99.58% (MIT-BIH) and 97.80% (Icentia11k). This architecture effectively integrates local feature extraction from CNNs and global sequence modeling from Transformers, ensuring both fine-grained temporal resolution and long-range dependency modeling. Across all metrics, including F1-score, Se, and PPV, the Hybrid Model consistently demonstrated superior performance across all classes.

By converting 1D signals into 2D time-frequency representations, the S-transform enables neural networks to extract both temporal and spectral features, significantly enhancing classification accuracy. CNNs excel at hierarchical and localized feature extraction, while Transformers model global dependencies across the 2D time-frequency domain^31^. The 2D Hybrid Model capitalizes on the strengths of both architectures, providing a robust and scalable solution for ECG analysis.

#### Analysis of correct and misclassified cases

**Fig. 5:**
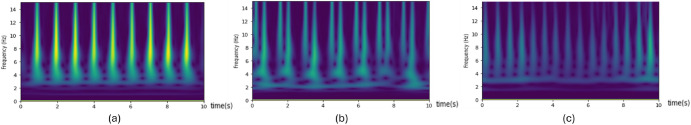
Analysis of correct and misclassified cases using S-transform spectrograms.

Figure 5a,b depict correctly classified cases. In Fig. 5a, all three spectrograms represent instances where the model correctly classified cases labeled as ‘N’ (Normal rhythm). The S-transform-based time-frequency spectrogram reveals regular and smooth energy distribution primarily in the 0-4 Hz range, which characterizes the N class. The proposed model effectively learned these regular patterns and accurately predicted the normal rhythm.

In Fig. 5b, two spectrograms show successful classification of cases labeled as ‘V’ (Ventricular arrhythmia). These spectrograms exhibit prominent rhythmic peaks in the 2-10 Hz frequency range, a distinct feature of V-class signals. Our model demonstrates its ability to capture these patterns and make precise predictions.

Figure 5c presents a misclassified case where the actual label is ’N’, but the model incorrectly predicted it as ’V’. The spectrogram shows a relatively consistent energy distribution in the 0-4 Hz range, typical of normal rhythms. However, there are noticeable frequency spikes in some segments that resemble V-class characteristics. These ambiguous patterns may have confused the model, leading to misclassification. Despite implementing techniques like Focal Loss and SE block, this case highlights the challenge of borderline signals. Further refinement, such as advanced preprocessing, data augmentation, or decision mechanisms, may help address such cases in the future.

In summary, the correctly classified cases in Fig. 5a,b highlight the model’s ability to learn stable and distinct spectral patterns for both N and V classes. Meanwhile, the misclassified case in Fig. 5c indicates potential ambiguities in certain signals that need to be mitigated with more sophisticated techniques.

#### Visualizing model interpretability in arrhythmia classification

**Fig. 6:**
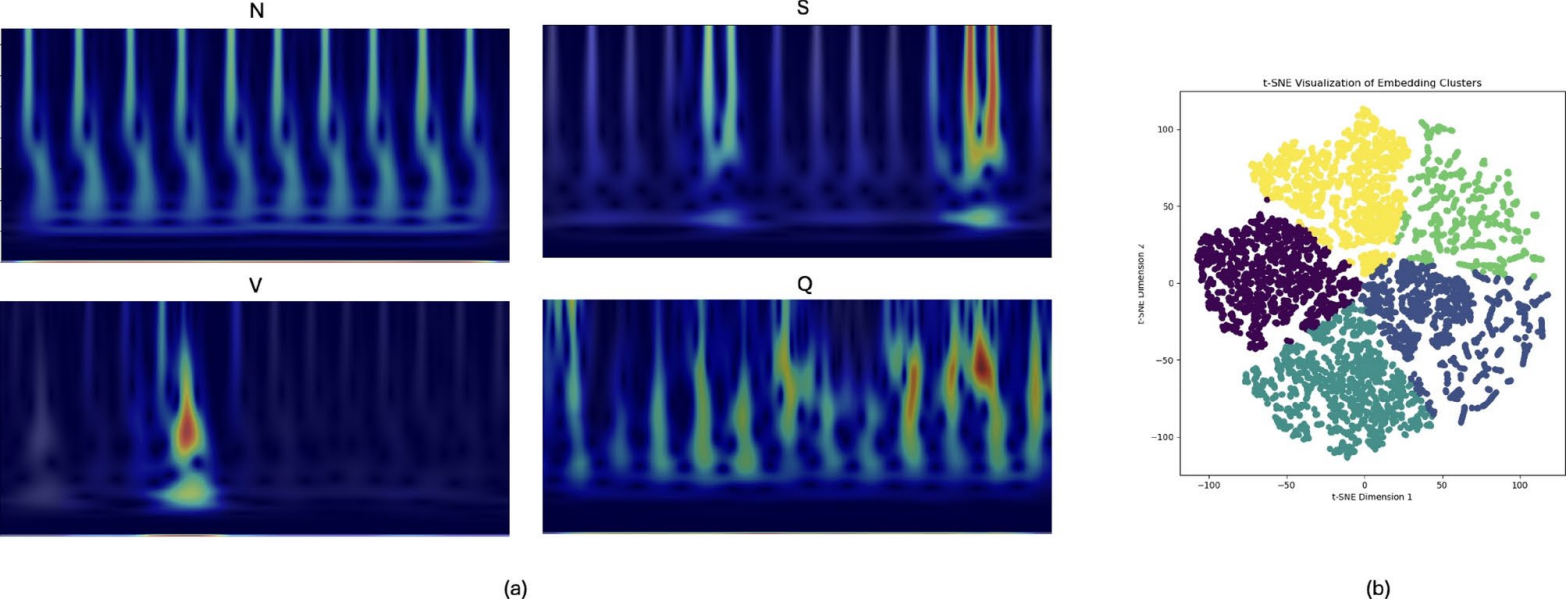
Model evaluation visualization: (**a**) CAM in the icentia11k dataset, (**b**) t-SNE visualization of the embedding clusters in the MIT-BIH dataset.

Figure 6a illustrates the Class Activation Maps (CAMs) for all arrhythmia classes (N, S, V, Q) in the Icentia11k dataset. These visualizations demonstrate how the model focuses on distinct time-frequency regions for each class during classification. The N class displays consistent low-frequency energy across time, while other arrhythmia classes exhibit variations such as localized mid-frequency peaks or dispersed patterns. These visualizations illustrate the interpretability of the model by revealing key regions that influence its decisions.

The t-SNE visualization in Fig. 6b depicts the embedding clusters for each arrhythmia label. Different colors in the figure correspond to specific arrhythmia classes. The embedding process was applied to the ECG data, and the resulting clusters illustrate the model’s effective separation of different arrhythmia types based on the learned feature space. The visualization emphasizes the clear grouping of similar arrhythmia patterns while also distinguishing between various distinct types. This figure demonstrates the model’s ability to capture the inherent structure within the data, resulting in meaningful clusters that reflect the underlying arrhythmia categories.

Through these ablation studies, we have developed a more profound comprehension of the interaction among different model components and their influence on performance. The findings have facilitated the refinement of the proposed model, guaranteeing that each component was configured to maximize its contribution to the overall effectiveness of the arrhythmia classification task.

#### Comparisons with classic methods

The comparison of our proposed model with several other methods documented in the literature is presented in Table 4. The results indicate that our model surpasses all prior methods in terms of *Acc*, *PPV*, and *Se*, establishing it as the superior classification model among those examined.

It is important to note that most existing methods do not utilize the entire dataset. For instance, some methods exclude the *N* or *F* classes and none incorporate the *Q* label, which hinders a fair comparison. In contrast, our method comprehensively evaluates and consistently achieves results across all classes. Specifically, the proposed model achieved an accuracy rate of 99.58% while incorporating all labels in the MIT-BIH database.

The model exhibits marginally lower efficacy in the N category compared to Fei-Yan’s model but demonstrates significant superiority in the S, V, and F categories, corresponding to crucial pathological conditions necessitating immediate medical attention^32^. This enhancement underscores the model’s proficiency in detecting severe arrhythmias, the misidentification of which could result in serious cardiac events. Consequently, notwithstanding a minor compromise in the N category, the model’s exceptional performance in identifying life-threatening arrhythmias offers substantial practical utility in clinical environments, where prompt and precise diagnosis is imperative for patient welfare.

**Table 4:** The performance of the proposed method compared to previous methods on the MIT-BIH dataset.

Methods	Acc(%)	N			S			V			Q			F		
		F1	Se	PPV	F1	Se	PPV	F1	Se	PPV	F1	Se	PPV	F1	Se	PPV
Lin et al.^33^	99.2	.962	.973	.951	.908	.905	.911	.982	.985	.979	–	–	–	.991	.983	1.00
Oliveira et al.^34^	95.3	.974	.971	.978	.916	.761	.566	.950	.930	.950	–	–	–	–	–	–
Chen et al.^35^	93.1	.969	.984	.954	.334	.295	.384	.773	.708	.851	–	–	–	–	–	–
Kung et al.^36^	98.6	–	–	–	.815	.754	.887	.970	.967	.974	–	–	–	–	–	–
Ince et al.^37^	98.3	–	–	–	.581	.635	.537	.860	.846	.874	–	–	–	–	–	–
Shi et al.^38^	94.2	.971	.953	.989	.627	.907	.479	.885	.929	.845	–	–	–	–	–	–
Xie et al.^39^	96.5	.900	.943	.877	.848	.797	.906	.966	.971	.962	–	–	–	.816	.908	.741
Zhai et al.^40^	96.1	.899	.879	.920	.754	.768	.740	.931	.938	.924	–	–	–	.700	.624	.796
Feiyan et al.^32^	**99.6**	**.999**	**.999**	**.999**	.973	.980	.966	.990	.991	.989	–	–	–	.924	.907	.942
Proposed	**99.6**	.997	.988	.991	**.997**	**.997**	**.997**	**.993**	**.994**	**.991**	**1.00**	**1.00**	**1.00**	**1.00**	**1.00**	**1.00**

Each class (N, S, V, Q, F) includes F1-score, Sensitivity (Se), and PPV. Significant values are in bold.

## Results

The utilization of the S-transform on ECG signals led to a significant enhancement in feature extraction and classification performance. Through providing a localized time-frequency representation, the S-transform facilitated the capture of both temporal and spectral information crucial for arrhythmia detection. This approach surpassed conventional methods such as Fourier and Wavelet Transforms by discerning subtle variations in ECG signals, resulting in higher classification accuracy. Moreover, the hybrid deep learning architecture, integrating CNNs for localized feature detection and Transformers for long-term dependency modeling, further bolstered the model’s capability. The model attained 97.8% accuracy on the Icentia11k dataset and 99.58% on the MIT-BIH dataset, outperforming previous methodologies. While the Icentia11k and MIT-BIH datasets primarily focus on common arrhythmias, which are generally easier to diagnose, this study lays the groundwork for applying the proposed deep learning model to more challenging cases. Future work will explore the model’s applicability to complex arrhythmias, such as atrial fibrillation and ventricular tachycardia. By extending the scope to include such conditions, this approach aims to assist cardiologists in addressing more difficult diagnostic challenges.

## Discussion

This research illustrates the effectiveness of the S-transform in enhancing ECG signal analysis, specifically for arrhythmia detection. The S-transform captured crucial temporal and spectral characteristics that conventional methods often overlook, resulting in superior classification accuracy. The hybrid CNN-Transformer architecture proved to be effective in analyzing both localized and long-term dependencies in the ECG signals, demonstrating robust performance on both the Icentia11k and MIT-BIH datasets. However, the study’s reliance on two datasets limits the generalizability of the findings, and future research should focus on testing the model across more datasets and noise conditions. Additionally, the computational complexity of the S-transform presents challenges for real-time analysis. Optimizing the model for speed while maintaining accuracy is a critical next step, particularly for deployment in clinical settings. Expanding generalizability and improving computational efficiency will be essential for broader clinical applications and real-time arrhythmia detection.

## Data Availability

The datasets analyzed in the present study were from the MIT-BIH Arrhythmia Database at https://physionet.org/content/mitdb/1.0.0/ and the Icentia11k database is available at https://physionet.org/content/icentia11k-continuous-ecg/1.0/. Accession codes: github
